# Promising biomedical applications using superparamagnetic nanoparticles

**DOI:** 10.1186/s40001-025-02696-z

**Published:** 2025-06-02

**Authors:** Yosri A. Fahim, Ibrahim W. Hasani, Waleed Mahmoud Ragab

**Affiliations:** 1 Health Sector, Faculty of Science, Galala University, Galala City, Suez 43511 Egypt; 2 Department of Pharmaceutics, Faculty of Pharmacy, S.P.U., M.P.U and Idlib University, Idlib, Syria; 3 Anatomy and Embryology Department, Faculty of Medicine, Galala University, Galala City, Suez 43511 Egypt

**Keywords:** Magnetic nanoparticles, Magnetic hyperthermia, Cancer therapy, Target drug delivery

## Abstract

Magnetic nanoparticles (MNPs) have emerged as powerful tools in biomedicine due to their distinct physicochemical characteristics, including a high surface-area-to-volume ratio, adjustable size, magnetic sensitivity, and compatibility with biological systems. These properties enable precise control through external magnetic fields, making MNPs highly effective in targeted therapeutic and diagnostic applications. Although not inherently intelligent, they can exhibit programmable and responsive behavior under external influence, enhancing their utility in drug delivery and hyperthermia-based treatments. In the medical field, MNPs have been extensively explored for their role in magnetic resonance imaging (MRI) enhancement, selective drug transport, hyperthermia cancer therapy, and biomolecular separation. Within oncology, they facilitate the direct delivery of therapeutic compounds to tumors, reducing systemic side effects and increasing treatment specificity. Additionally, their capacity to produce localized heat when exposed to alternating magnetic fields makes them instrumental in hyperthermia therapy, where malignant cells are selectively eradicated. A key advantage of MNPs is their adaptable surface chemistry, which allows for functionalization with biocompatible polymers, ligands, and other stabilizing agents. These modifications enhance their stability, minimize immune responses, and optimize their performance in physiological environments. Functionalized MNPs have contributed significantly to improving MRI contrast, refining drug delivery mechanisms, and increasing the effectiveness of hyperthermia treatments. This review examines recent breakthroughs in MNP-based medical technologies, with an emphasis on tumor targeting, drug delivery across the blood–brain barrier, and hyperthermia applications.

## Highlights

- MNPs can be controlled with magnetic fields for targeted treatments and imaging.
- MNPs improve cancer therapy by delivering drugs directly to tumors with minimal side effects.
- Surface functionalization enhances MNPs' stability, imaging, and drug delivery efficiency in biomedical use.

## Introduction

Nanoparticles (NPs) are materials characterized by at least one dimension within the nanometer scale, typically up to approximately 100 nm [1]. Due to their high surface-area-to-volume ratio, NPs exhibit unique physicochemical properties that differ from their bulk counterparts, making them highly versatile for various scientific and industrial applications [2]. Among these, MNPs have gained substantial attention due to their responsiveness to external magnetic fields, making them valuable in diverse fields such as biomedicine, catalysis, environmental remediation, and energy storage [3]. They are typically composed of metal-based structures, including iron, cobalt, nickel, titanium, and ferrite compounds like magnetite (Fe₃O₄) and maghemite (γ-Fe₂O₃). These materials exhibit superparamagnetic behavior, meaning they can rapidly transition between magnetic states upon exposure to an external magnetic field, driven by their magnetic moment and field properties, and lose their magnetization when the field is removed, behaving as nonmagnetic particles [4]. This property, along with their high specific absorption rate (SAR) under alternating magnetic fields (AMF), enables efficient heat generation for applications like hyperthermia therapy, enhancing treatment outcomes in cancer therapy through material composition and structural optimization [5, 6]. The physicochemical properties of these particles, including their size, surface charge, colloidal stability, and biocompatibility, significantly influence their potential applications [7]. While MNPs, such as iron oxide-based nanoparticles, exhibit inherent stability in physiological environments, modifications such as surface functionalization with biocompatible polymers (e.g., dextran, polyethylene glycol) or inorganic coatings (e.g., silica, gold) further enhance their stability, prevent aggregation, and optimize their interaction with biological systems[2]. Recent studies have demonstrated that optimizing colloidal stability through appropriate surface modifications prevents nanoparticle aggregation, ensuring their effective circulation in biological environments [8, 9]. Additionally, improving biocompatibility through functionalization minimizes immune system activation, reducing cytotoxic effects and enhancing therapeutic efficacy [10]. Beyond superparamagnetism, properties like high saturation magnetization and anisotropic behavior further dictate MNPs’ suitability for medical and industrial applications [11]. In biomedicine, magnetic nanoparticles have been extensively researched for their role in magnetic resonance imaging (MRI) contrast enhancement, targeted drug delivery, hyperthermia therapy, and biosensing [12]. Moreover, MNPs have shown significant promise in the field of theranostics, a rapidly evolving approach that integrates therapeutic and diagnostic capabilities into a single nanoscale platform. In this context, MNPs can be engineered to act both as imaging agents, such as contrast enhancers in MRI, and as therapeutic carriers for drug delivery or hyperthermia treatments [13]. The theranostic application of MNPs not only enhances treatment precision, but also minimizes systemic toxicity, making them a key component in the advancement of personalized and targeted medicine [14]. MNPs’ capacity to react to external magnetic fields facilitates accurate localization within the body, minimizing systemic adverse effects in therapeutic applications [15]. They have demonstrated significant potential in clinical medicine, particularly in targeted therapies for cancer treatment, neurological disorders, and infectious diseases [16]. Their use in hyperthermia therapy for cancer, where localized heating induced by alternating magnetic fields selectively destroys malignant cells, has shown promising results. Additionally, functionalized MNPs have facilitated advancements in drug delivery systems by enabling site-specific release and controlled drug administration [17].

Additional limitations or requirements are largely determined by the intended use of the MNPs, whether in vitro or in vivo. In vitro applications typically have fewer constraints compared to in vivo ones [18]. For magnetic structures, a biocompatible polymer coating is often essential if the nanoparticles lack inherent biocompatibility. This coating, applied during or after manufacturing, helps reduce toxicity, minimize the risk of blood capillary constriction, and prevent nanoparticle aggregation [2]. The continuous evolution of nanotechnology has paved the way for groundbreaking advancements in magnetic nanoparticle research. By refining synthesis methods, optimizing surface modifications, and enhancing biocompatibility, MNPs hold immense promise for revolutionizing biomedical applications. However, challenges related to large-scale production, toxicity, and regulatory approval must be addressed before their full clinical potential can be realized.

This review explores the developments in MNP-based technologies, focusing on their synthesis, characterization, properties, and biomedical applications. Additionally, it highlights the challenges and future perspectives of integrating them into clinical practice, aiming to bridge the gap between laboratory research and real-world medical applications.

## Synthesis methods of magnetic nanoparticles

**Fig. 1:**
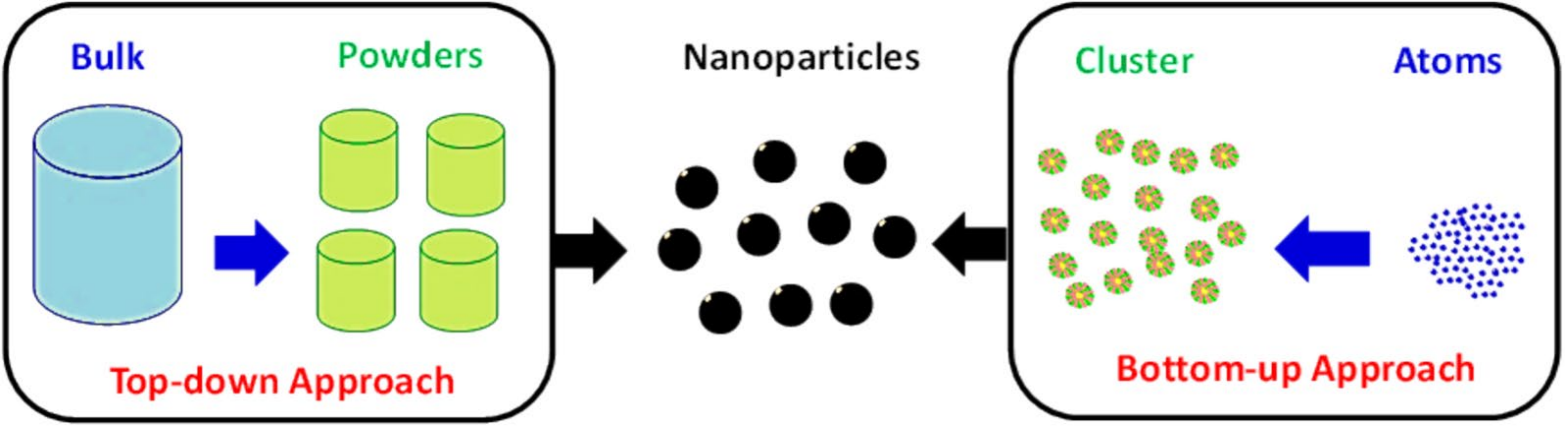
Synthesis techniques of nanoparticles

Nanoparticles (NPs) can be synthesized using two primary techniques: top-down and bottom-up approaches, as illustrated in Fig. 1.

**Fig. 2:**
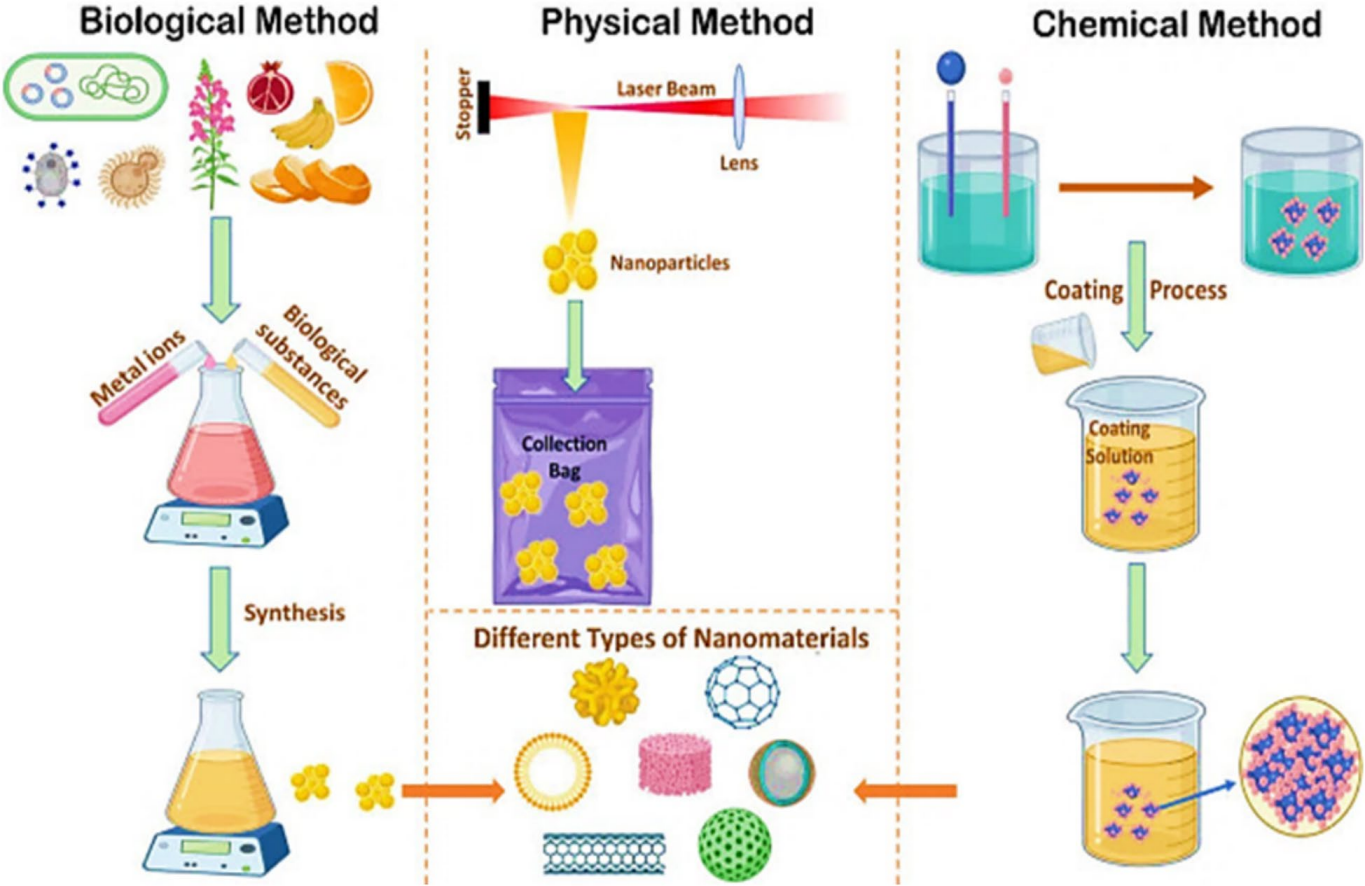
Methods for synthesizing various types of MNPs [19]

The synthesis of MNPs is a critical factor influencing their size, shape, crystallinity, magnetic behavior, and biocompatibility [2]. Various methods have been developed to produce them with controlled physicochemical properties, broadly categorized into chemical, physical, and biological approaches; Fig. 2 [19].

### Chemical methods

Chemical synthesis is the most widely used approach for producing monodisperse, high-purity with tailored surface characteristics. Some of the most common chemical techniques include:

- Co-precipitation: A simple, cost-effective method where ferrous (Fe^2^⁺) and ferric (Fe^3^⁺) salts undergo precipitation in an alkaline medium, forming iron oxide nanoparticles (Fe₃O₄ or γ-Fe₂O₃) [20, 21]. *Laurent *et al*.* illustrated its efficacy in synthesizing superparamagnetic iron oxide nanoparticles (SPIONs) for MRI, highlighting the significance of pH and ionic strength in regulating particle size [22]. While scalable, it often results in polydisperse particles and requires post-synthesis modifications for enhanced stability.
- Thermal decomposition: Organometallic precursors decompose at high temperatures in organic solvents with surfactants, yielding highly crystalline and monodisperse nanoparticles [23]. Sun et al*.* refined this methodology, attaining exact size control (4–20 nm) for Fe₃O₄ nanoparticles suitable for biological applications [24]. This method allows precise control over particle size and shape but involves complex synthesis conditions and organic solvents, limiting biocompatibility.
- Microemulsion: A surfactant-stabilized nanoscale reaction environment that controls nucleation and growth, producing uniform MNPs [25]. However, it has a low yield and requires extensive purification. Spinel ferrites are generated in microemulsions and inverse micelles. For example, MnFe_2_O_4_ nanoparticles with diameters ranging from 4 to 15 nm are generated using water-in-toluene inverse micelles and NaDBS as a surfactant [26].
- Sol–gel method: Involves the hydrolysis and condensation of metal precursors, producing highly stable magnetic nanostructures [27]. It is widely used for coating magnetic cores with silica or other protective layers. Ferrites like CoFe₂O₄ (15–40 nm) are produced with metal nitrates and citric acid, offering tunable magnetic properties after calcination at 600–800 °C [22]. Hybrid MNPs (e.g., Fe₃O₄@TiO₂, 20–60 nm) combine magnetic and photocatalytic traits [27].
- Hydrothermal and solvothermal synthesis: Conducted under high temperature and pressure in a sealed autoclave, yielding high-purity, crystalline nanoparticles with excellent colloidal stability [28]. Li et al*.* synthesized monodisperse, hydrophilic, single-crystalline ferrite microspheres using hydrothermal reduction [29]. Wang et al. found that a 40-nm nanoscale Fe_3_O_4_ powder can be made using the hydrothermal technique at 140 °C for 6 h, with a saturation magnetization of 85.8 emu. g^−1^ [30].
- Electrochemical synthesis: Uses electrolysis of metal salts to generate nanoparticles with precise size and shape control but is limited by low production rates [31].

### Physical methods

Physical methods rely on energy-intensive techniques to produce MNPs by reducing bulk materials to nanoscale dimensions or directly forming nanoparticles from precursors [32]. These methods include:

- Ball milling: A mechanical grinding process that reduces bulk magnetic materials into nanosized particles [33, 34]. While scalable, it often leads to broad particle size distributions and irregular morphologies. For example, iron nanoparticles smaller than 50 nm have been created via prolonged milling of iron powder in an argon atmosphere [35].
- Laser ablation: A high-energy laser beam vaporizes a metal target in a liquid or gas medium, forming pure, contaminant-free MNPs with controlled morphology [36]. For instance, iron oxide nanoparticles (Fe₃O₄) with sizes ranging from 5 to 30 nm have been synthesized using pulsed laser ablation of an iron target in water, demonstrating high purity, superparamagnetic behavior, and suitability for biomedical applications such as MRI contrast enhancement and hyperthermia therapy [37].
- Sputtering: A vacuum-based deposition method where high-energy ions bombard a target material, releasing nanoparticles with controlled thickness and structure [38]. Copper (Cu) nanoparticles, averaging 10–20 nm in size, were produced using DC magnetron sputtering. The resultant nanoparticles demonstrated elevated purity and a limited size distribution, making them appropriate for catalytic applications [39].

### Biological methods

Biological synthesis offers an eco-friendly, non-toxic approach for their synthesis using microorganisms, plant extracts, or biomolecules as reducing and stabilizing agents [40].

- Microbial synthesis: Certain bacteria, such as *Magneto spirillum* species, naturally produce magnetosomes biogenic MNPs with precise size and shape control. These nanoparticles exhibit excellent biocompatibility and potential for medical applications [41]. *Pseudomonas aeruginosa* produces extracellular iron oxide MNPs (10–30 nm) in iron-rich, carbon-minimal media at low pH, with magnetic alignment confirmed via external fields [40].
- Plant-mediated synthesis: Plant extracts containing bioactive compounds (phenols, flavonoids, tannins) reduce metal precursors to form biocompatible and functionalized MNPs without toxic chemical reagents [42]. For example, zinc ferrite nanoparticles were synthesized using guava leaves extract for biomedical applications [1].
- Enzyme-assisted synthesis: Enzymes such as laccases and reductases mediate MNP formation, allowing for highly controlled synthesis under mild conditions [43]. Nitrate reductases from microbial sources catalyze the reduction of Fe^3^⁺ to Fe^2^⁺, forming iron oxide MNPs (e.g., magnetite, Fe₃O₄) with sizes of 5–50 nm [43]. While hydrogenase enzymes from *Clostridium pasteurianum* reduce Fe^3^⁺ intracellularly to form magnetite MNPs (~ 15 nm) [41].

## General structure of magnetic nanoparticles

Magnetic nanoparticles consist of a magnetic core responsible for their magnetism and a protective surface layer that enhances stability, prevents aggregation, and allows for functionalization [44]. Their structural properties are influenced by composition, crystal arrangement, and surface modifications, which determine their applications in biomedicine, electronics, and environmental science. They can be synthesized from a variety of compositions and phases, encompassing pure metallic elements, metal oxides (e.g., Fe₃O₄, γ-Fe₂O₃), ferrites (e.g., BaFe₁₂O₁₉, SrFe₁₂O₁₉, and MFe₂O₄, where M represents divalent cations such as Cu, Ni, Mn, Mg), and metal alloys (e.g., CoPt, FePt). While iron oxide nanoparticles are the most widely studied, other materials, such as ferrites, metallic nanoparticles, and hybrid core–shell structures, also exhibit significant magnetic properties and are used in various fields [45].

### Iron oxide nanoparticles

Iron oxide-based NPs, including Fe₃O₄ and γ-Fe₂O₃, are commonly used due to their biocompatibility, superparamagnetic behavior, and stability in physiological environments. These materials have a spinel crystal structure, where oxygen ions form a face-centered cubic (FCC) lattice while iron ions occupy both tetrahedral and octahedral sites. In Fe₃O₄, iron exists in a mixed Fe^2^⁺/Fe^3^⁺ state, whereas in γ-Fe₂O₃, Fe^3^⁺ ions dominate, with some vacancies in the crystal lattice. These structural differences influence their magnetic response and oxidation resistance [46].

Iron oxide nanoparticles are frequently employed in MRI, drug delivery, and hyperthermia therapy due to their ability to be manipulated under an external magnetic field. However, they tend to form aggregates, which is why their surfaces are often modified with polymeric (PEG, dextran), inorganic (silica, gold), or biological (antibody, peptide) coatings to enhance dispersion and improve functionality [47].

### Ferrite-based nanoparticles

Ferrites are mixed metal oxides with the general formula MFe₂O₄, where M represents divalent cations such as manganese (Mn), cobalt (Co), nickel (Ni), or zinc (Zn). These nanoparticles exhibit either ferrimagnetic or superparamagnetic behavior, depending on their size and composition [48].

- Cobalt ferrite (CoFe₂O₄) has high magnetic anisotropy and chemical stability, making it suitable for high-density data storage and magnetic sensors.
- Nickel ferrite (NiFe₂O₄) exhibits moderate magnetization and electrical conductivity, which makes it useful in biomedical imaging and catalysis.
- Manganese ferrite (MnFe₂O₄) has tunable magnetic properties and is often employed in drug delivery and hyperthermia applications.
- Zinc ferrite (ZnFe₂O₄) is a low-magnetization material commonly used in magnetic fluids and sensing devices.

Ferrite nanoparticles are advantageous due to their stability, resistance to oxidation, and ability to be synthesized with controlled magnetic properties. To further enhance their performance, they are often coated with biopolymers, silica, or surfactants for improved dispersibility in aqueous environments [22].

### Metallic magnetic nanoparticles

In addition to metal oxides and ferrites, pure metals such as iron (Fe), cobalt (Co), nickel (Ni), and metal alloys (e.g., FePt, CoPt) exhibit superior magnetic properties compared to metal oxide-based nanoparticles. These properties make them ideal for magnetic storage, electromagnetic shielding, and catalysis. However, pure metal nanoparticles are prone to oxidation and corrosion, necessitating protective coatings or alloying with more stable elements [49]. Below are examples of metallic magnetic nanoparticles.

- Iron nanoparticles possess strong ferromagnetic behavior, but are highly reactive to oxygen and moisture. They are often coated with carbon, silica, or noble metals to improve stability.
- Cobalt and nickel nanoparticles exhibit high coercivity and strong magnetic interactions but can be cytotoxic in biological applications, limiting their use unless encapsulated in inert coatings.
- Iron–platinum and cobalt–platinum nanoparticles are known for their exceptional chemical stability and high magnetic anisotropy, making them ideal for biomedical imaging and high-performance magnetic storage.
- Rare-earth magnetic materials, such as samarium–cobalt (SmCo) and neodymium–iron–boron (NdFeB), provide strong permanent magnetism, which is useful in industrial and electronic applications.

Due to their superior magnetization properties, metallic nanoparticles are often used in high-performance applications but require surface modifications for practical use in biomedical or environmental settings [50].

### Core–shell and hybrid magnetic nanoparticles

**Fig. 3:**
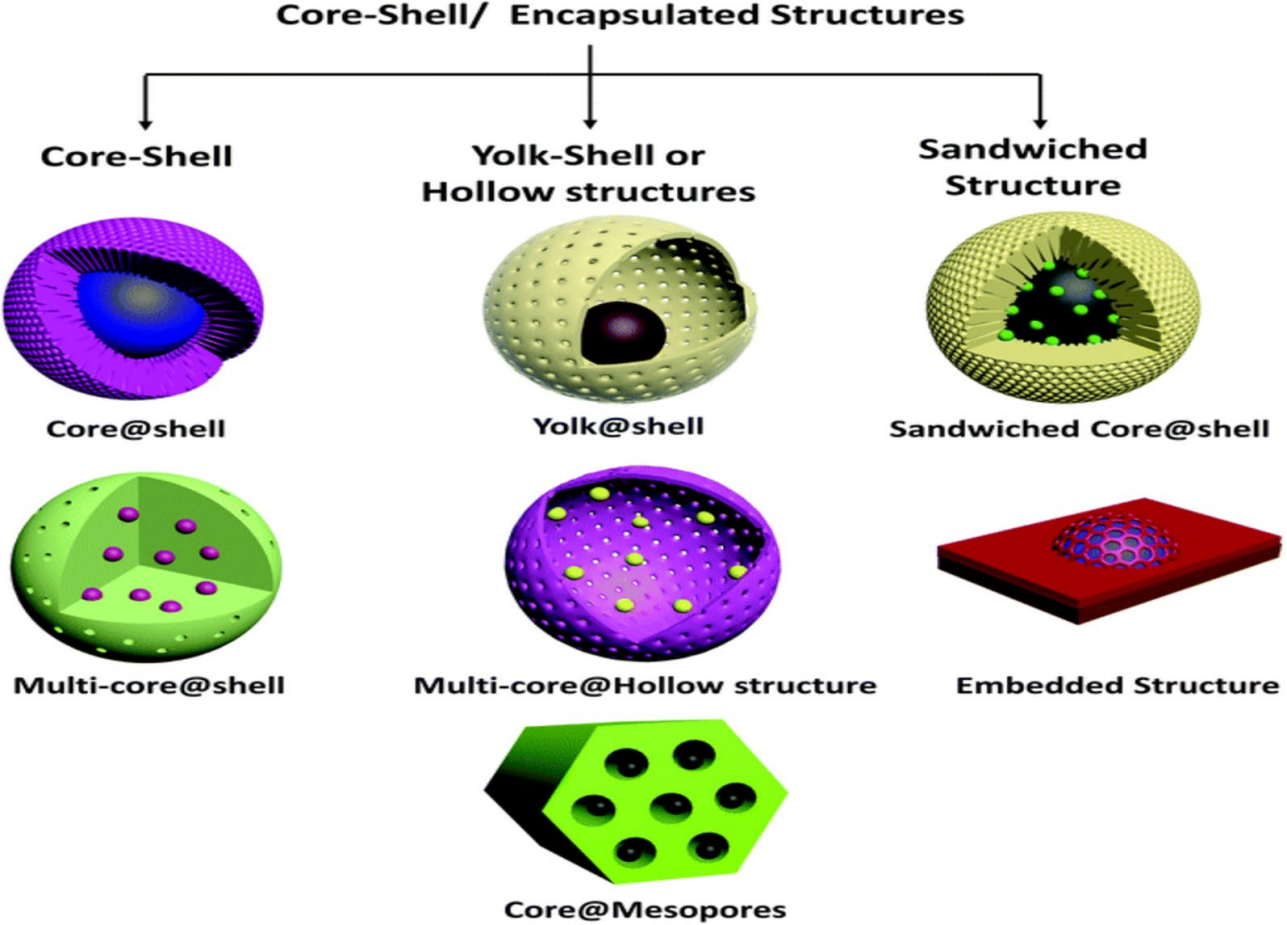
Core–shell sample of a metal core coated with silica or polymers [52]

To enhance the functionality of MNPs, core–shell structures have been developed, where a magnetic core is surrounded by a protective shell [51]. This design improves biocompatibility, dispersibility, and chemical stability while allowing for functionalization with targeted molecules; Fig. 3 [52].

The core–shell structure consists of two distinct layers: the magnetic core, which governs the nanoparticle’s magnetic properties, and the shell, which serves as a protective barrier or functional layer [53]. Several core–shell configurations have been developed, each offering unique advantages:

- Silica-coated MNPs (Fe₃O₄@SiO₂) provide excellent chemical stability and biocompatibility, making them ideal for biosensing and drug delivery.
- Gold-coated MNPs (Fe₃O₄@Au) combine magnetic and plasmonic properties, which are useful in bioimaging and theranostic.
- Polymer-coated nanoparticles enhance colloidal stability and reduce toxicity, which is essential for medical applications.
- Carbon-coated MNPs improve electrical conductivity and oxidation resistance, making them valuable for energy storage and catalysis.

## Advantages of magnetic nanoparticles in medical applications

### Size

The biomedical efficacy of MNPs is closely linked to their dimensions, specifically two parameters: core size and hydrodynamic diameter (HD), which influence several functional aspects. The core size, usually assessed using transmission electron microscopy (TEM), influences the magnetic characteristics of MNPs, including superparamagnetism and saturation magnetization, which are essential for applications such as hyperthermia treatment and MRI [11]. For example, MNPs with core diameters between 10 and 50 nm demonstrate excellent magnetic responsiveness, balancing robust magnetization and superparamagnetic properties to inhibit aggregation without an external magnetic field [54]. Conversely, the hydrodynamic diameter (HD), including the core and surface coatings (such as polymers or silica) and generally evaluated using dynamic light scattering (DLS) in physiological environments, dictates their pharmacokinetics and biodistribution in vivo [2]. In this section, size references relate to HD unless stated differently. MNPs having a hydrodynamic diameter (HD) between 10 and 100 nm provide an ideal equilibrium of extended circulation duration, effective cellular internalization, and advantageous biodistribution. Renew filtration swiftly eliminates parts with a hydrodynamic diameter of less than 10 nm, limiting their retention and therapeutic efficacy [55].

In contrast, particles larger than 200 nm are susceptible to detection and elimination by the mononuclear phagocyte system (MPS), especially in the liver and spleen, heightening the risk of accumulation and toxicity [56]. Nanoparticles with a 30 to 50 nm hydrodynamic diameter are superior for MRI contrast enhancement due to their substantial surface-area-to-volume ratio and increased relativity, enhancing imaging resolution [57].

In drug delivery, MNPs in the 50–100 nm range can efficiently penetrate biological barriers and leverage the enhanced permeability and retention (EPR) effect to accumulate in tumor tissues while avoiding rapid immune clearance [15]. However, the EPR effect remains highly controversial, with some studies suggesting it may be overstated or even a fallacy in certain contexts due to tumor heterogeneity, inconsistent vascular permeability, and limited clinical translation in humans compared to preclinical models [58, 59]. Similarly, hyperthermia therapy benefits from particles sized 10–50 nm, as they demonstrate superior heat dissipation, efficiently converting electromagnetic energy into localized heating [60]. Additionally, surface modifications such as polymer or silica coatings can alter their hydrodynamic size, affecting their circulation and targeting efficiency. Precise control over nanoparticle size enables researchers to optimize pharmacokinetics, cellular interactions, and therapeutic efficacy, ensuring their safe and effective application across various medical fields [61].

### Remote control functionality

MNPs exhibit remote control functionality due to their ability to respond to external magnetic fields. This property allows for precise modulation of their movement and aggregation, which is particularly valuable in biomedical applications such as targeted drug delivery and biological labeling [62]. The interaction with external magnetic fields occurs through two key mechanisms: the gradient effect and alternating magnetic fields. The gradient effect relies on spatial variations in the magnetic field to guide them to specific locations within the body [63]. This targeted approach enhances drug delivery efficiency by concentrating therapeutic agents at the intended site while minimizing systemic exposure and side effects. Alternating magnetic fields, on the other hand, induce localized heating of MNPs, which can be leveraged for hyperthermia treatment in oncology. Additionally, the controlled movement of MNPs enables their application in precise biological labeling, where they can be guided to specific cells or tissues for imaging and diagnostic purposes [64].

### Resonance reaction to field variations

Under alternating magnetic fields (AMF), MNPs undergo rapid magnetization reversals, dissipating energy as heat for applications such as hyperthermia therapy [65]. This localized heating is a key mechanism in magnetic hyperthermia, selectively destroying cancerous cells while sparing healthy tissues [66]. The extent of this thermal effect is influenced by factors such as nanoparticle composition, size, and the frequency of the applied magnetic field. Beyond hyperthermia, resonance-based magnetic interactions are also being explored for applications in controlled drug release and enhanced imaging contrast in MRI diagnostics [67]. To contextualize these advantages, Table 1 compares MNPs with other nanoparticle types commonly used in biomedical applications.

**Table 1:** A comparative analysis between magnetic nanoparticles and other nanoparticles in biomedical applications

Type	Advantages	Limitations
Magnetic nanoparticles (MNPs)	• Magnetic responsiveness for targeting and hyperthermia • High surface-area-to-volume ratio for functionalization • Heat generation for cancer therapy • Versatile (MRI, drug delivery, hyperthermia)	• Potential cytotoxicity from metal cores • Complex synthesis for uniformity • Aggregation risk without modification • Limited drug loading capacity
Gold nanoparticles (AuNPs)	• Plasmonic properties for imaging and photothermal therapy • High biocompatibility and stability • Easy functionalization via thiol chemistry	• No magnetic control for targeting • Expensive, limiting scalability • Limited drug encapsulation • Size-dependent toxicity
Liposomes	• High drug encapsulation capacity • Excellent biocompatibility • Versatile for various agents (e.g., drugs, genes) • Controlled release via triggers	• No magnetic targeting • Stability issues (leakage, degradation) • Short circulation time without modification • Complex production
Dendrimers	• Precise structure for controlled drug loading • Multifunctional with multiple attachment sites • High solubility for hydrophobic drugs	• No magnetic properties • Complex, costly synthesis Potential toxicity • Limited scalability
Carbon nanotubes (CNTs)	• Large surface area for drug loading • Photothermal properties for therapy • High cellular uptake • Useful in biosensing and tissue engineering	• Potential cytotoxicity and poor solubility • No magnetic targeting • Difficult purification and synthesis • Biocompatibility concerns without functionalization
Silica nanoparticles (SiNPs)	• High biocompatibility and stability • Tunable porosity for drug loading • Easy surface functionalization • Used in imaging and drug delivery	• No magnetic properties • Limited targeting without modification • Potential long-term accumulation in organs • Lower therapeutic versatility

## Magnetic nanoparticle in drug delivery process

MNPs play a crucial role in drug delivery by enabling precise targeting, controlled release, and enhanced therapeutic efficacy [68]. This section outlines the stages of MNP-based drug delivery, ensuring relevance to their biomedical applications.

### Drug loading

Magnetic nanoparticles can be engineered with various coatings and functional groups to enhance drug loading efficiency and stability. Encapsulation strategies include covalent bonding, electrostatic interactions, and polymeric coatings, which prevent premature drug release and improve bioavailability [69].

### Circulation and targeting

**Fig. 4:**
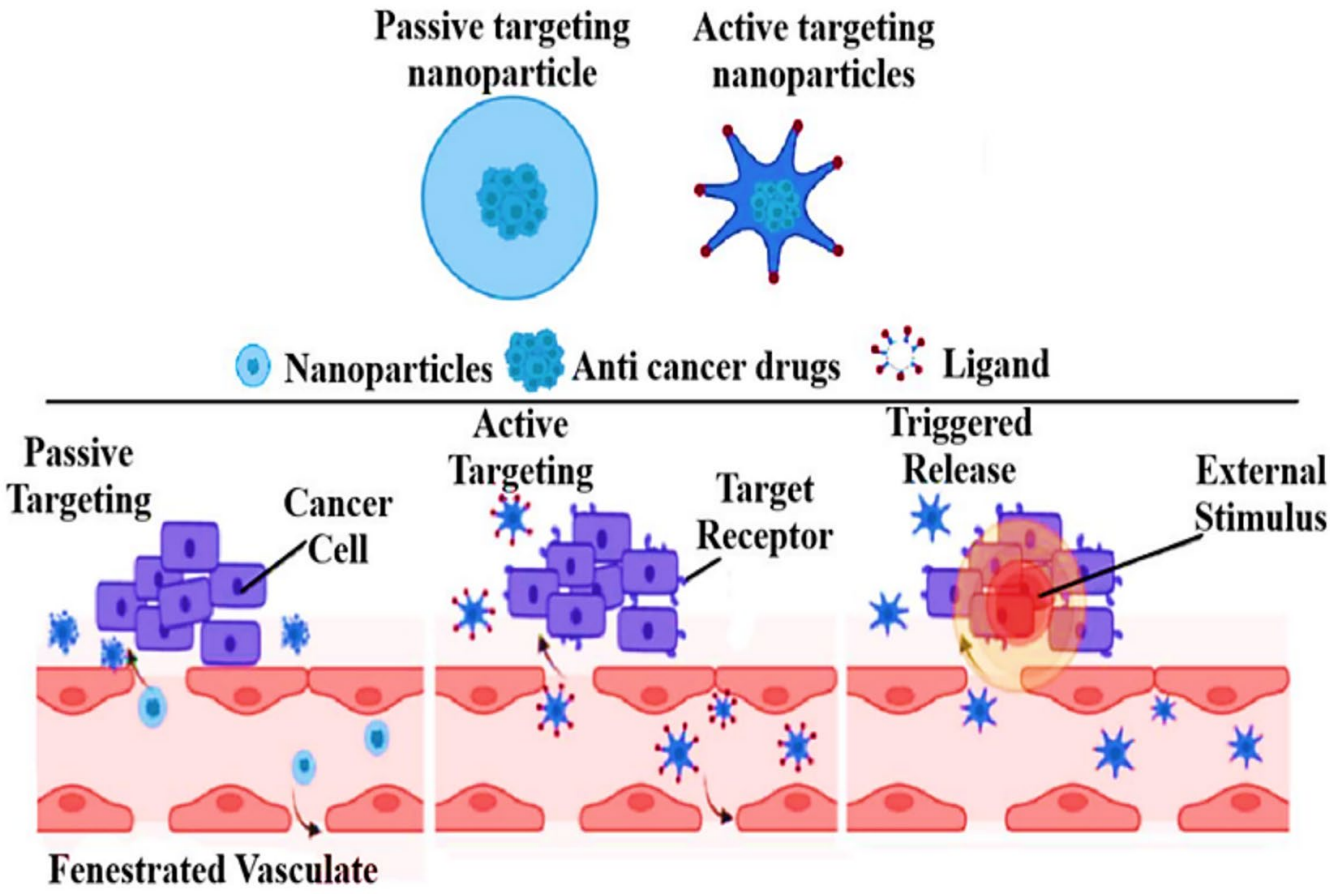
Active and passive targeting of nanoparticles [71]

Upon administration, MNPs travel through the bloodstream, with their distribution influenced by size, surface modifications, and interactions with biological components. Their ability to evade immune clearance is essential for prolonged circulation [70]. They reach target tissues via passive or active targeting mechanisms; Fig. 4 [71].

Passive targeting: utilizes the enhanced permeability and retention (EPR) effect to concentrate MNPs in tumour areas, attributed to compromised vasculature and inadequate lymphatic drainage [72]. The dependability of the EPR effect varies among tumor types, species, and stages, potentially overestimating its efficacy for metal-based nanoparticles. This method is significantly influenced by nanoparticle size, shape, and surface characteristics [61], but it often leads to generalized accumulation in the tumor microenvironment rather than selective uptake by malignant tumor cells [73].

Active targeting: Conversely, it enhances the precision of magnetic nanoparticle (MNP) drug delivery by using ligands such as antibodies, peptides, or small molecules that bind to specific receptors either on tumor cells or within the tumor microenvironment [74]. This approach can be categorized into tumor cell-specific targeting, which involves receptors overexpressed on malignant cells, and tumor tissue-specific targeting, which focuses on receptors located on nonmalignant cells such as endothelial or stromal cells within the tumor environment. For example, folic acid-functionalized MNPs bind to folate receptors commonly overexpressed on cancer cells, facilitating direct delivery to tumor cells [75]. In contrast, transferrin-coated MNPs target transferrin receptors on endothelial cells of the blood–brain barrier, promoting accumulation in brain tumor regions without directly targeting tumor cells [76]. Targeting integrins on tumor vasculature is another tissue-level strategy, promoting broader accumulation but potentially reducing cancer cell specificity. The distinction between these targeting types is vital: tissue-level targeting aids accumulation in the tumor microenvironment, while cell-level targeting ensures precise delivery to malignant cells. Challenges like ligand stability, receptor heterogeneity, and off-target binding can affect both strategies’ accuracy [77].

### Controlled drug release and therapeutic action

Magnetic nanoparticles enable precise drug release through external magnetic stimuli, utilizing various controlled release mechanisms. One approach is magnetic hyperthermia, where an alternating magnetic field generates localized heat, triggering drug release and inducing apoptosis in cancer cells. Another strategy involves pH-sensitive coatings that degrade in the acidic tumor microenvironment, ensuring site-specific drug activation. Additionally, enzyme-responsive systems rely on specific enzymatic activity at the disease site to initiate drug release, further enhancing targeted therapeutic efficacy [78].

### Biocompatibility and clearance

The biocompatibility and clearance of MNPs are essential considerations for their safe and effective clinical use. The mononuclear phagocyte system (MPS) plays a key role in recognizing and eliminating nanoparticles based on their size and surface characteristics. In the context of drug delivery, optimizing factors such as hydrodynamic diameter, surface charge, and coating materials (e.g., polyethylene glycol (PEG), dextran, or silica) is critical for minimizing immune recognition and improving systemic circulation [79]. Surface modifications help minimize opsonization, thereby decreasing MPS uptake and enhancing biodistribution. Neutral or slightly negative surface charges are generally associated with reduced macrophage internalization compared to positively charged particles, which tend to interact more strongly with cell membranes [80]. Advanced coatings such as zwitterionic polymers can further prevent protein corona formation and promote extended circulation times [81]. Additionally, the use of biodegradable materials supports safe elimination through renal or hepatic pathways, lowering the risk of long-term accumulation and associated toxicity [79]. These strategies underscore the importance of rational nanoparticle design to maximize biocompatibility and therapeutic performance.

## Applications of magnetic nanoparticles in biomedical research

**Fig. 5:**
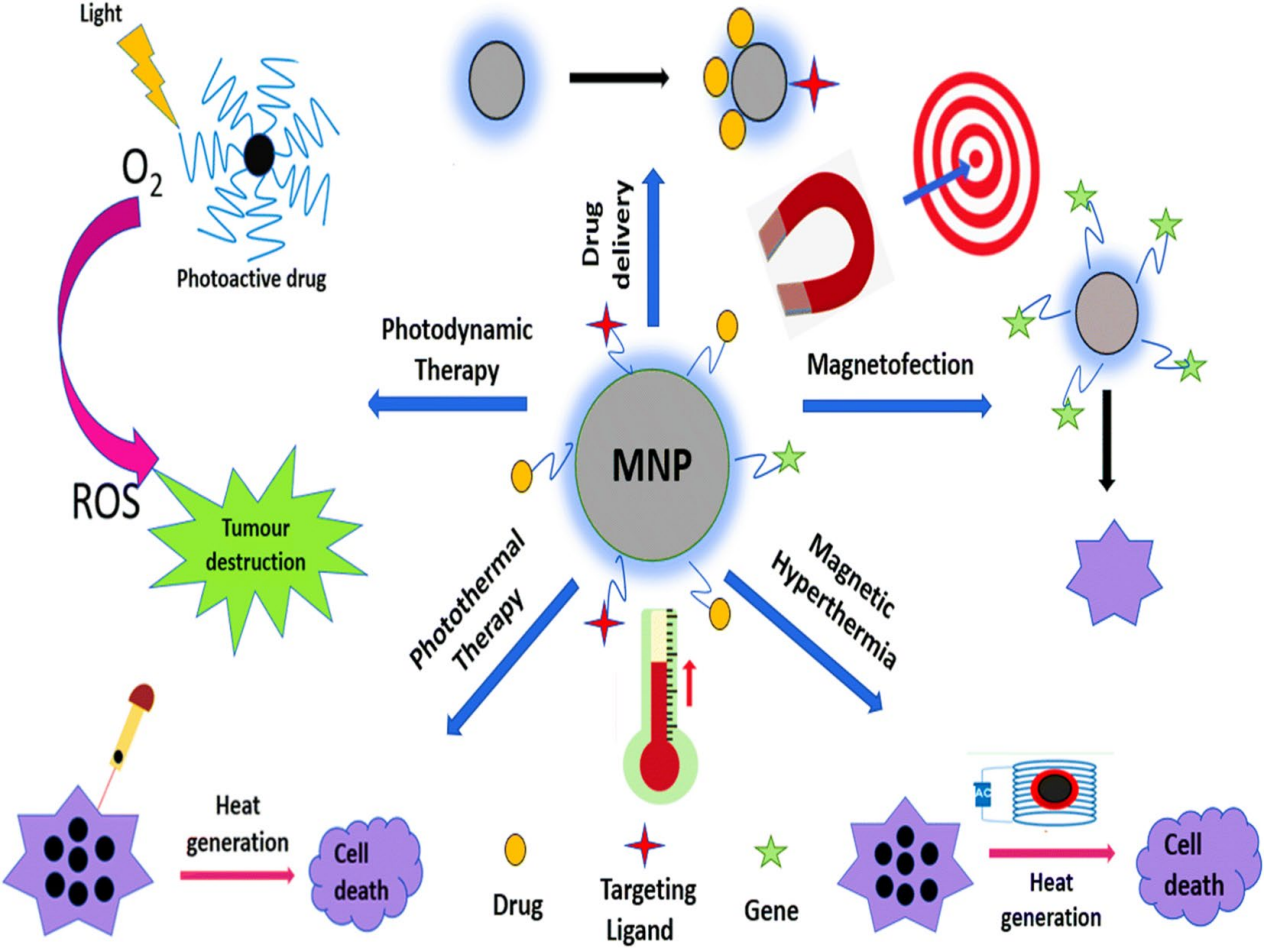
Applications of MNPs in biomedical research [82]

Magnetic nanoparticles have a broad range of applications in biomedical research, demonstrating significant potential in areas such as medical imaging, targeted drug delivery, and regenerative medicine. Their ability to be guided and manipulated using external magnetic fields makes them valuable tools for precise and non-invasive therapeutic interventions; Fig. 5 [82].

### Magnetic resonance imaging (MRI)

MNPs enable advanced MRI applications, including cancer imaging, in vivo tracking of iron oxide-doped stem cells, and monitoring transplanted tissue functionality, offering precise insights into disease progression and therapeutic outcomes [83]. The application of superparamagnetic magnetite nanocrystal clusters (SMNCs) in cellular imaging represents a valuable and versatile tool in biomedical research [84]. These SMNCs exhibit remarkable magnetic resonance sensitivity, maintain cell viability without adverse effects, and demonstrate properties that are both dose- and time-dependent [85, 86]. Superparamagnetic iron oxide nanoparticles (SPIONs) have been extensively employed as tracers in preclinical models for the treatment of cardiovascular and central nervous system disorders [87]. Notably, iron oxide nanoparticles (IONPs) functionalized with polyethylene glycol (PEG) markedly improve blood circulation durations, diminish immune clearance, and facilitate extended MRI monitoring of biodistribution, essential for monitoring therapeutic administration and tissue targeting [87]. Zhao et al. investigated the viability and proliferation of bone marrow mesenchymal stem cells labeled with superparamagnetic iron oxide nanoparticles (SPIONs). Their findings revealed that delivering cell transplants via liver or spleen injections could accelerate liver regeneration. Furthermore, in vivo MRI of SPIONs-labeled cells enables real-time monitoring of liver regeneration, offering valuable insights for clinical management post-hepatectomy [88]. A summary clinical application of various types of MNPs is provided in Table 2.

**Table 2:** Investigational biomedical applications of various types of magnetic nanoparticles

Magnetic nanoparticles	Methods of synthesis	Specific diseases targeted	Mechanisms of action	Clinical applications	Refs.
Gold-coated MNPs (Fe₃O₄@Au)	Thermal decomposition, co-precipitation	Glioblastoma, breast cancer	Photothermal ablation via plasmonic heating, targeted drug release	Theranostics targeted drug delivery, and photothermal therapy	[89]
Silica-coated MNPs (Fe₃O₄@SiO₂)	Sol–gel method	Liver cancer, brain tumors	Enhanced MRI contrast, controlled drug release via porous structure	Biosensors, drug delivery systems, and enhanced MRI imaging	[90]
Polymer-coated MNPs	Co-precipitation, emulsion polymerization	Breast cancer, pancreatic cancer	Magnetic hyperthermia, biocompatible drug encapsulation and release	Controlled drug delivery, biocompatible imaging agents, and hyperthermia	[91]
Magnetic nanoclusters (e.g., Fe₃O₄ clusters)	Thermal decomposition	Solid tumors (e.g., melanoma)	Collective magnetic heating, enhanced MRI sensitivity	Enhanced imaging and hyperthermia due to collective magnetic properties	[24]
Cerium-doped iron oxide nanoparticles (Ce-doped Fe₃O₄)	Hydrothermal synthesis	Osteosarcoma, lung cancer	ROS modulation, radiosensitization via cerium doping	Radiotherapy enhancement and reactive oxygen species (ROS) modulation for cancer therapy	[92]
Plasmonic-MNPs	Laser ablation, chemical reduction	Skin cancer, prostate cancer	Plasmonic heating for hyperthermia, enhanced optical imaging	bioimaging and hyperthermia	[12]
Magnetite–gold hybrid nanoparticles (Fe₃O₄–Au)	Thermal decomposition	Glioblastoma, colorectal cancer	Dual imaging (magnetic and X-ray absorption), photothermal cell destruction	Dual imaging (MRI and X-ray CT), photothermal therapy, and drug delivery	[93]
Lanthanide-doped MNPs (Ln-doped Fe₃O₄)	Solvothermal synthesis	Brain tumors, lymphomas	Luminescent signaling for imaging, magnetic targeting	Luminescence-based bioimaging combined with MRI	[94]
Calcium ion-doped magnesium ferrite nanoparticles	Hydrothermal synthesis	Breast cancer, liver cancer	Photothermal heating enhanced by calcium doping	Photothermal therapeutic materials for cancer treatment	[95]
Mn–Zn ferrite nanocrystals	Microemulsion	Hepatocellular carcinoma	Magnetically induced hyperthermia targeting cancer cells	Magnetically–induced cancer targeted hyperthermia	[96]
Mn–Zn ferrite MNPs	Co-precipitation	Lung cancer, pancreatic cancer	Synergistic hyperthermia and radiosensitization	Enhancing targeted cancer treatment by combining hyperthermia and radiotherapy	[97]
Cupric oxide nanoparticles (CuO NPs)	Biological synthesis (plant-mediated)	Embryonic trophoblast-related conditions	Induction of cell death via oxidative stress	inducing embryonic trophoblast cell death	[98]
Zinc oxide nanoparticles loaded with calendula extract	Biological synthesis (plant extract)	Burn wounds	Anti-inflammatory and wound healing via calendula, ROS scavenging	Burn wound healing	[99]
Cobalt ferrite nanoparticles (CoFe₂O₄)	Hydrothermal synthesis	Diabetes, cardiovascular diseases	Magnetic signal amplification for biosensing	Magnetic biosensors for detecting diseases like diabetes or cardiovascular disorders	[11]
Cubic-shaped cobalt ferrite nanoparticles (Co–Fe NCs)	Solvothermal synthesis	Melanoma, breast cancer	High-anisotropy magnetic heating for hyperthermia	Serve as magnetic hyperthermia agents	[100]

### Magnetic nanoparticles in cancer therapy and chemotherapy

Cancer poses a significant challenge to society, with a growing global incidence affecting millions of individuals [101]. Magnetic nanoparticles have emerged as powerful tools in cancer management, integrating targeted drug delivery, advanced imaging, and innovative therapies to improve outcomes [102]. In chemotherapy, MNPs enhance precision by conjugating anticancer agents such as doxorubicin, gemcitabine, or methotrexate to their surfaces, allowing external magnetic fields to guide them to tumor sites [103]. Modified with biocompatible coatings (e.g., dextran, polyethylene glycol), functional ligands, or antibodies, MNPs achieve selective interaction with malignant cells, facilitating controlled drug release via magnetic modulation, pH shifts, or enzymatic triggers [104]. In cancer chemotherapy, this approach enables sustained accumulation of anticancer drugs (e.g., doxorubicin) at tumor sites, enhances cellular uptake via receptor-mediated endocytosis, and reduces systemic toxicity, thereby improving therapeutic outcomes [74]. Beyond chemotherapy, MNPs enhance cancer diagnostics and imaging by leveraging their superparamagnetic properties to improve tumor visualization, aiding early detection and treatment planning [105].

Multifunctional MNPs enable a theranostic approach, combining therapy and real-time monitoring, as seen with platforms integrating drug delivery and MRI contrast enhancement. Additionally, they expand treatment options through gene therapy and immunotherapy, acting as vectors for genetic material or immune stimulants, offering alternatives to heat-based methods [106]. Table 3 outlines the diverse applications of MNPs in drug delivery and cancer therapy.

**Table 3:** Applications of MNPs in drug delivery and cancer therapy

Drug name	Nanoparticle	Targeted cancer	Biological pathway	Refs.
Acyclovir	Fe_3_O_4_ MNPs	Brain cancer	Inhibition of viral replication in tumor cells	[107]
Doxorubicin	Gelatin/Fe_3_O_4_-alginate	Breast cancer	Induction of apoptosis via DNA intercalation	[108]
Doxorubicin	Magnetic iron oxide NPs (MIONs)	Liver cancer	ROS-mediated oxidative stress and apoptosis	[109]
Doxorubicin (DOX)	Iron oxide nanoparticles	Breast cancer	Tumor cell killing via (magnetic hyperthermia and chemotherapy)	[110, 111]
Erlotinib	Mesoporous MNPs/folic acid	Lung cancer	Inhibition of EGFR signaling pathway	[112]
Methotrexate	Chitosan-coated Fe_3_O_4_	Ovarian cancer	Folic acid receptor-mediated endocytosis	[113]
Gemcitabine	Fe_3_O_4_, metformin, and peptide pHLIP	Pancreatic cancer	Disruption of tumor metabolism and apoptosis	[114]
Telmisartan	Fe3O4/chitosan	Prostate cancer	Angiotensin receptor blockade and cell cycle arrest	[115]
Chemo/hyperthermia therapy	Tragacanth gum/polycrylic acid/Fe_3_O_4_ nanoparticles	Colorectal cancer	Synergistic effect of hyperthermia and chemotherapy	[116]
Gene therapy	Fe_3_O_4_/polyethyleneimine (PEI)	Leukemia	Enhanced gene transfection and targeted therapy	[117]
Radiation therapy	Au/iron oxide	Various solid tumors	Enhancement of radiation sensitivity through localized hyperthermia	[118]
Zidovudine	NiFe_2_O_4_/poly (ethylene glycol)/lipid NPs	Lymphoma	Inhibition of viral replication and tumor progression	[119]
Doxorubicin	Porous carbon-coated Fe_3_O_4_ nanoparticles	Melanoma	Heat-induced apoptosis and enhanced drug delivery	[120]

### Magnetic nanoparticles in hyperthermia treatment

**Fig. 6:**
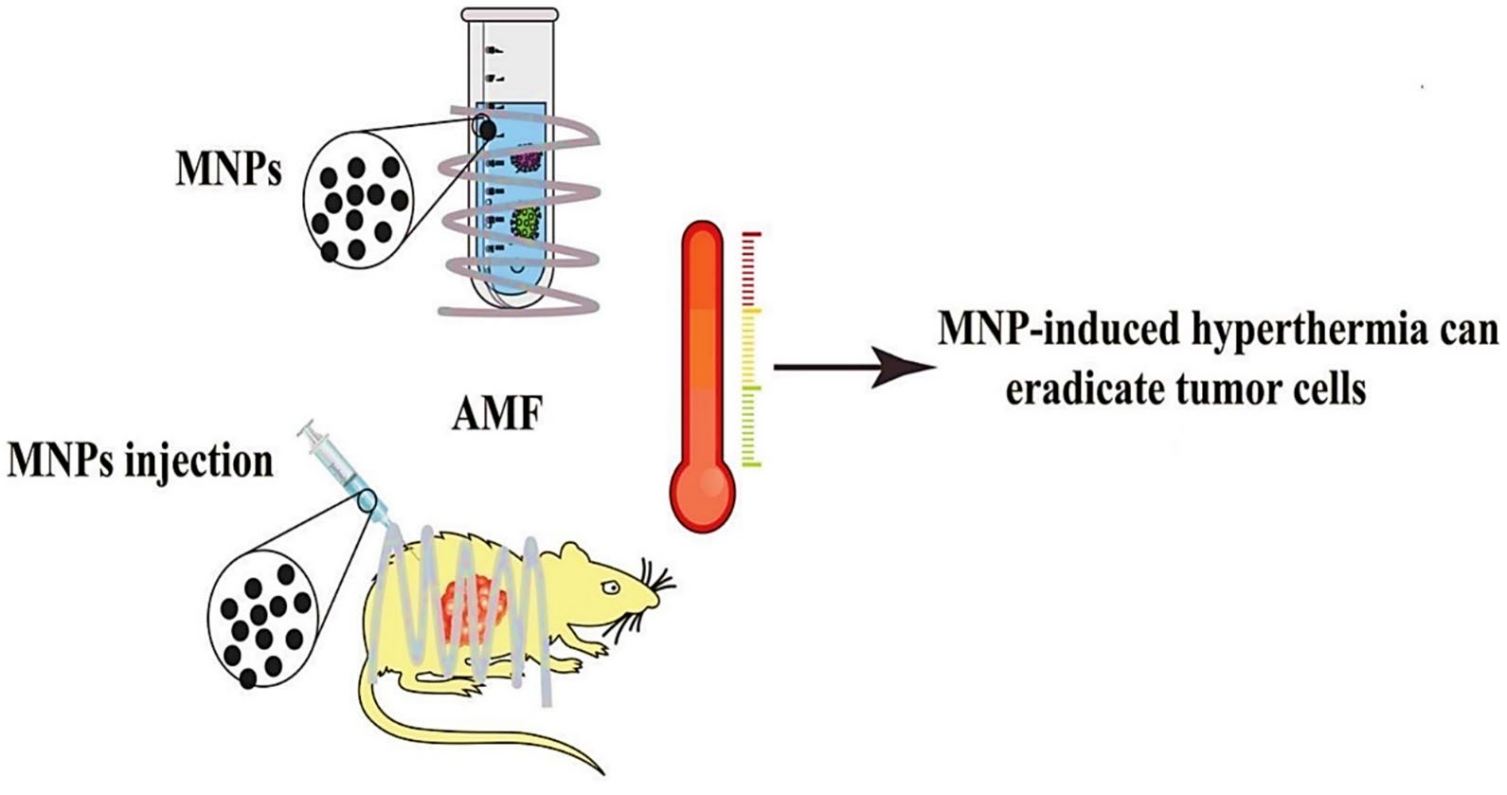
Magnetic hyperthermia therapy (MHT) [121]

Magnetic hyperthermia is an innovative cancer treatment that utilizes heat generated by MNPs to selectively destroy tumor cells. When exposed to an alternating magnetic field (AMF), they produce localized heat through Néel and Brownian relaxation mechanisms, raising the temperature within the tumor microenvironment to 42–46 °C; Fig. 6 [121].

This mild hyperthermia disrupts cancer cell function, induces apoptosis, and enhances tumor responsiveness to chemotherapy and radiation therapy. Notably, *MagForce AG*’s *NanoTherm*^*®*^ therapy, the only clinically approved magnetic nanoparticle-based hyperthermia therapy, utilizes superparamagnetic iron oxide nanoparticles (SPIONs) for glioblastoma treatment, approved by the European Medicines Agency (EMA) as a medical device [122]. SPIONs, widely studied for their biocompatibility and efficient heat generation, are intratumorally injected and activated by an external alternating magnetic field, generating localized heat that selectively damages cancer cells while minimizing systemic side effects compared to whole-body hyperthermia [123]. In contrast, advanced targeting strategies, such as glucuronic acid-functionalized iron oxide nanoparticles (IONPs) for glioblastoma, employ intravenous administration to exploit systemic circulation and GLUT-mediated transcytosis, augmented by moderate hypoglycemia, achieving unparalleled success in active targeting [124]. High-anisotropy materials like cobalt ferrite (CoFe₂O₄) nanoparticles, often intratumorally administered, demonstrate superior heating capabilities in both in vitro and in vivo studies for cancers like melanoma and breast cancer, while iron–platinum (FePt) nanoparticles show promise primarily in vitro [100].

Intratumoral injection ensures high nanoparticle concentrations at the tumor site, enhancing heating efficiency and minimizing systemic exposure, but it is invasive and limited to accessible, localized tumors [125]. Intravenous administration enables systemic delivery to metastatic or inaccessible tumors, leveraging active or passive targeting, but faces challenges such as MPS clearance, variable EPR effects, and potential off-target accumulation [126]. These limitations highlight the need for optimized nanoparticle design and targeting strategies to balance efficacy and safety. While intratumoral injection dominates in vivo magnetic hyperthermia studies, intravenous administration is gaining attention for its potential to target metastatic or inaccessible tumors [127]. To enhance tumor targeting, magnetic nanoparticles can be functionalized with ligands, antibodies, or specific molecules like glucuronic acid to improve cancer cell recognition and uptake [124]. Spinel ferrites, particularly effective in breast cancer treatment, exhibit superior efficacy in vitro and in vivo compared to traditional therapies [128]. Hyperthermia is frequently combined with chemotherapy, immunotherapy, or radiotherapy to maximize treatment outcomes, leveraging the synergistic effects of localized heating and other modalities [129].

### Photodynamic therapy

Photodynamic therapy (PDT) is an innovative cancer treatment that utilizes light-sensitive compounds, known as photosensitizers, which, upon activation by specific wavelengths of light, generate reactive oxygen species (ROS) to induce targeted cytotoxicity [130, 131]. However, the effectiveness of PDT is often limited by the poor solubility of photosensitizers, off-target effects, and inefficient tumor accumulation [132]. MNPs have been introduced as promising carriers to overcome these limitations, facilitating enhanced delivery and precise activation of photosensitizers. MNPs in PDT offer several advantages, including improved solubility, stability, and controlled targeting of photosensitizers. By conjugating photosensitizers to the surface of these nanoparticles or encapsulating them within biocompatible coatings, advanced systems have been developed to enhance tumor accumulation through both passive and active targeting [133]. In PDT, photosensitizers primarily produce ROS to induce cytotoxicity, distinct from photothermal therapy (PTT), where heat generation is the primary mechanism [12]. However, MNP–photosensitizer hybrids can integrate PDT with hyperthermia, where MNPs generate heat via alternating magnetic fields or, in some cases, photothermal effects when combined with plasmonic materials like gold [134]. Recent advancements in MNP-mediated PDT focus on optimizing light penetration depth, engineering nanoparticles with improved photostability, and exploring external stimuli-responsive systems that allow precise control over photosensitizer activation [81].

### Magnetic nanoparticle for drug delivery

**Fig. 7:**
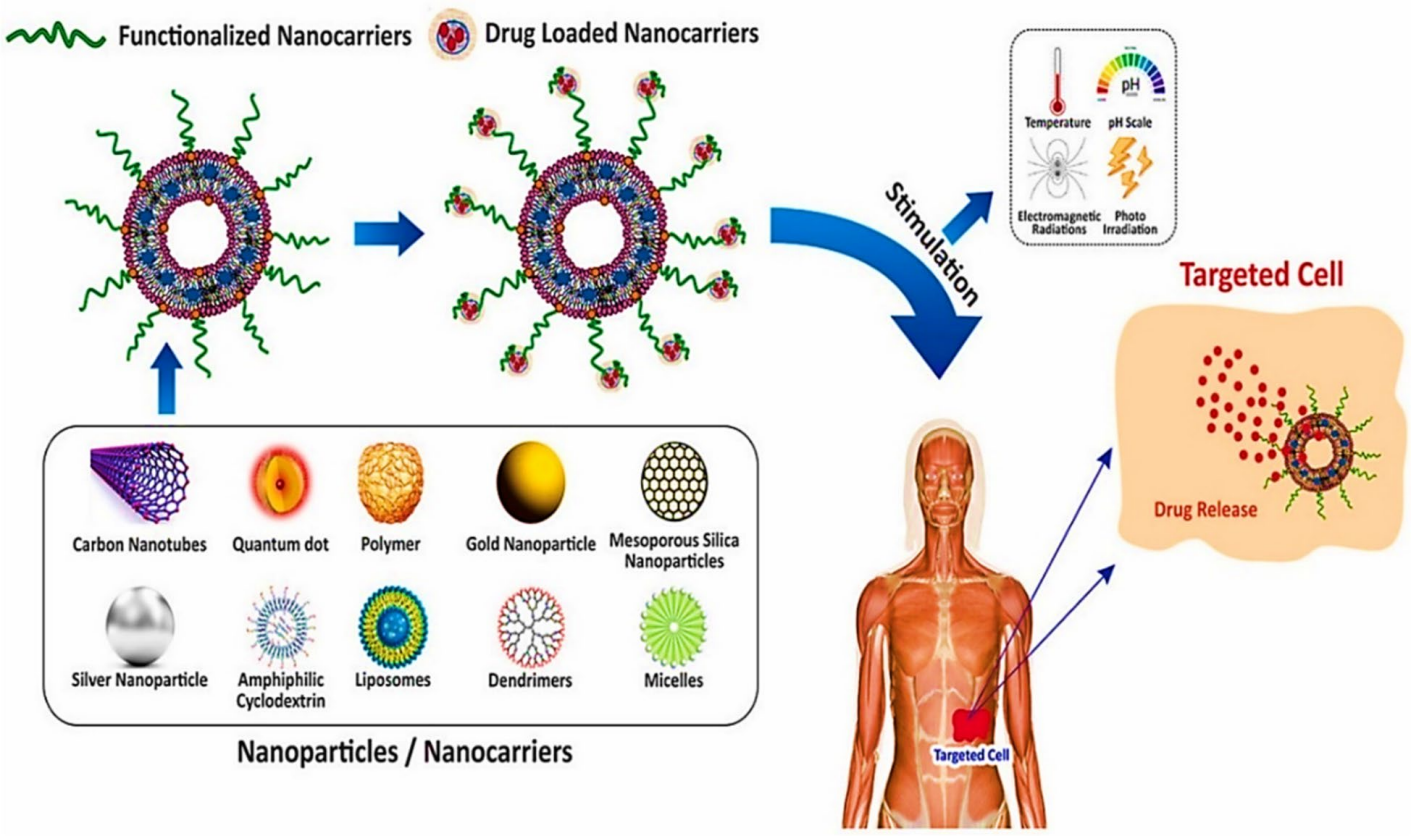
Types of targeted drug delivery system [136]

Magnetic nanoparticles offer a powerful strategy for targeted drug delivery, utilizing external magnetic fields to direct therapeutic agents precisely to diseased tissues [102]. Unlike PDT, where magnetic nanoparticles deliver photosensitizers that generate reactive oxygen species upon light activation to induce cell death, drug delivery systems using magnetic nanoparticles focus on transporting therapeutic agents and releasing them at targeted sites in response to external stimuli such as pH shifts, magnetic fields, or enzymatic activity [135]. Here, the therapeutic effect is derived from the drug itself rather than from light-triggered photochemical reactions. This approach enhances drug accumulation at specific sites, minimizing systemic distribution and reducing side effects while improving overall therapeutic efficacy. Biocompatible coatings, such as dextran, chitosan, polyethylene glycol (PEG), or silica, enhance MNP stability and drug conjugation efficiency, optimizing targeted delivery. Additionally, controlled drug release can be achieved through external stimuli such as pH changes, enzymatic activity, or heat application via magnetic hyperthermia; Fig. 7 [136].

Superparamagnetic iron oxide nanoparticles (SPIONs) are among the most widely used materials due to their biocompatibility, strong magnetic responsiveness, and ability to enable targeted drug release. These nanoparticles have been extensively investigated for cancer therapy, delivering chemotherapeutic agents such as doxorubicin, paclitaxel, and methotrexate directly to tumor sites [137]. When functionalized Fe₃O₄ nanoparticles are combined with chemotherapy and hyperthermia, treatment precision is significantly improved, ensuring localized drug release and reducing toxicity to healthy tissues [138]. Beyond oncology, MNP-based drug delivery has shown promise in combating bacterial infections by acting as carriers for antimicrobial agents rather than exhibiting direct antibacterial effects. Various antimicrobial compounds, including TiO₂, ZnO, MgO, silver, and chitosan, have been conjugated with them to enhance their localized therapeutic action [139]. Silver-coated magnetic beads were successfully guided by an external magnetic field to inhibit *E. coli* growth, demonstrating improved antibacterial activity at targeted sites. This strategy enhances bacterial eradication while minimizing systemic exposure to antimicrobial agents, thereby reducing the risk of antibiotic resistance. Furthermore, the nanoscale size of them increases their surface area-to-volume ratio, leading to a higher drug-loading capacity and more controlled antibiotic release, making them an effective tool for treating drug-resistant infections [140].

### Radioimmunotherapy

Radioimmunotherapy employs low doses of radioactive isotopes to treat cancer, utilizing antibodies to specifically target and accumulate radionuclides within tumors, ultimately destroying malignant cells through sustained radiation emission [141]. Recently, SPIONs and other nanocarriers have been identified as potential radionuclide delivery systems [142]. Despite its potential, radioimmunotherapy has demonstrated limited success in treating solid tumors, likely due to restricted accessibility and variable tumor radiosensitivity. Unlike chemotherapeutics, the use of SPIONs in radiotherapy poses challenges, as the continuous decay of radioactive isotopes can hinder the protection of healthy cells [143]. As a result, after cellular uptake, SPION-radionuclide complexes must remain stable until the radiation decays to prevent exposure to non-target cells. The most commonly used radioactive isotope for creating radionuclide-SPIONs is 188Re, which has a half-life of 17 h and has been shown to induce cell death primarily in targeted hepatic cells in vivo [144].

### Gene therapy and magnetofection

Gene therapy involves the introduction of foreign DNA into a patient to treat or correct a genetic disorder [145]. The introduced DNA usually includes a functional gene that reduces the impact of a pathogenic mutation [146]. Significant progress has been made, particularly in gene transfer and expression technologies, with an emphasis on developing safer, more durable gene vectors and enhancing cell specificity. Antisense RNA can be used in gene therapy to block the expression of defective genes [147]. This groundbreaking discovery has the potential to enhance a wide range of applications, including genetic disorders, cardiovascular diseases, cancer, and neurological conditions. Developing an efficient gene delivery system that targets and integrates plasmid DNA into specific genomic loci is crucial for the success of gene therapy. Despite extensive research, optimal results in gene therapy remain elusive due to several challenges. These include a lack of specificity, the limited lifespan of genes in vivo, and poor diffusion across cell membranes, resulting in relatively low transfection efficacy and difficulties in targeting specific locations without causing harmful or detrimental side effects [148]. Magnetofection is a technique that combines MNPs with nonviral or viral vectors to enhance gene delivery when exposed to a magnetic field [149]. Using MNPs as vectors for antisense oligodeoxynucleotides (ODNs) can help address many of the challenges mentioned earlier. when combined with gene therapies, serve as gene delivery vectors that protect nucleic acids from enzymatic degradation and promote endosomal release after cellular uptake [150]. MNP-based transfection provides several advantages, including the need for lower vector doses, versatility as both viral and nonviral vectors, and high-efficiency transfection with short incubation periods. Furthermore, this method allows for gene delivery to difficult-to-transfect cells and enables precise in vivo targeting, making it a valuable tool for genetic research and therapy. These benefits position MNPs as a promising solution to overcome challenges in gene delivery systems [151]. Stable, nontoxic MNP-gene vector complexes enable the use of magnetic gene targeting (MGT) in suitable animal models.

### Biosensors

A biosensor is a device that converts a biological event into a measurable and easily detectable signal [152]. MNPs are ideal platforms for biosensors due to their easily modifiable surfaces, which can accommodate various receptors, and their ability to function as ferromagnetic labels [153]. They can also disperse within the sample and be used on the active detection surface of the biosensor. They are extensively employed in sensing applications, particularly for directly labeling substrates on sensors in conjunction with transducer materials [154]. Various biosensors are designed to detect specific biological molecules. *Chen *et al*.,* employed MNPs and a dual-marker identification approach to create a highly sensitive biosensor based on surface plasmon resonance spectroscopy [155]. The cytosensor shows great potential for exploring new applications in detecting various separation products from MNPs. Gold and MNPs have been used to develop a multilayered polymeric DNA sensor that utilizes radio frequency technology [156].

### Tissue engineering

Magnetic nanoparticles have gained interest in tissue engineering due to their ability to enhance scaffold properties, promote cellular interactions, and enable remote-controlled stimulation [157]. While various nanomaterials have been explored for skeletal repair, only those with intrinsic or functionalized magnetic properties are relevant in this context. Iron oxide-based nanoparticles are widely used in regenerative medicine due to their superparamagnetic behavior, biocompatibility, and ability to influence cellular activity under magnetic fields. They can be incorporated into biocompatible scaffolds to enhance osteogenesis, facilitate cell proliferation, and guide tissue regeneration [158]. Although materials like forsterite nanopowder, nanoTiO₂, fluorapatite, and TiO₂-based nanocomposites have been explored for bone regeneration due to their biocompatibility and bioactivity, they lack inherent magnetic properties [159]. However, functionalizing these materials with MNPs enables their use in magnetically assisted tissue engineering applications. For example, magnetic nanocomposites combining hydroxyapatite with Fe₃O₄ have been shown to enhance bone regeneration and accelerate healing through magnetically guided cell differentiation [160]. Additionally, copper-enhanced multi-walled carbon nanotubes (MWCNTs) have been investigated for antibacterial and mechanical reinforcement applications in implants. While they do not exhibit magnetism in their pure form, their integration with MNPs has been explored for multifunctional biomaterials, combining antimicrobial, mechanical, and magnetic-responsive properties [161].

## Cytotoxicity of magnetic nanoparticles

Magnetic nanoparticles hold great promise for biomedical applications. However, ensuring their biocompatibility is crucial, as their potential cytotoxic effects could impact clinical safety. Several factors influence MNP cytotoxicity, including size, shape, surface chemistry, composition, dose, and exposure duration [2]. Smaller nanoparticles can penetrate cells more easily, potentially causing toxicity, while surface coatings and functional groups play a critical role in stability and immune interactions. The metal core and magnetic properties of MNPs can also contribute to cellular stress, and excessive aggregation may alter their biological interactions [80]. Notably, the route of administration (e.g., intravenous, inhalation, or topical) can further modulate these effects, as it determines the nanoparticles’ interaction with different biological barriers and tissues [162]. The mechanisms underlying MNP-induced cytotoxicity include oxidative stress, membrane disruption, mitochondrial dysfunction, inflammatory responses, and potential genotoxic effects [163]. Oxidative stress occurs when reactive oxygen species (ROS) are generated, leading to lipid peroxidation, protein oxidation, and DNA damage [164]. Direct interactions with cell membranes can compromise structural integrity, while mitochondrial dysfunction may reduce ATP production and induce apoptosis. Additionally, They can trigger immune activation and inflammation, which may contribute to adverse effects at the tissue level [165]. For instance, uncoated iron oxide nanoparticles have been shown to induce higher ROS levels compared to polymer-coated variants, highlighting the protective role of surface modifications [166]. Furthermore, chronic exposure to MNPs may lead to cumulative effects, such as organ-specific toxicity in the liver or spleen, where nanoparticles tend to accumulate [167]. To mitigate these cytotoxic effects, various strategies have been developed. Surface modification with biocompatible polymers such as polyethylene glycol (PEG), chitosan, or dextran enhances stability and minimizes immune recognition. Optimizing dosage and exposure duration through rigorous in vitro and in vivo studies helps establish safe concentration thresholds. The incorporation of biodegradable materials promotes nanoparticle clearance, reducing long-term accumulation and toxicity risks [166]. Functionalizing with specific targeting ligands can improve drug delivery precision, minimizing off-target effects. Additionally, advanced coatings, such as zwitterionic polymers, have emerged as a promising approach to reduce protein corona formation and enhance circulation time, further lowering toxicity [81]. Additionally, standardized toxicological assessments, including cell viability assays, oxidative stress evaluations, and genotoxicity studies, are essential for ensuring the safety of MNPs before clinical use [167]. These assessments should also account for real-world variables, such as patient-specific factors (e.g., age, immune status) and the intended therapeutic context, to better predict clinical outcomes [79]. Table 4 below summarizes key factors influencing MNP cytotoxicity.

**Table 4:** Key factors influencing MNP cytotoxicity

Factor	Biological impact	Refs.
Size	Smaller sizes increase cellular uptake, potentially causing toxicity	[2]
Surface chemistry	Uncoated surfaces trigger immune responses	[80]
Dose/exposure	High doses or prolonged exposure induce stress	[165]
Composition	Metal cores (e.g., Fe, Co) may release toxic ions	[163]
Aggregation	Aggregates disrupt cellular function	[166]

## Challenges and future perspectives

Magnetic nanoparticles hold great potential in biomedicine, environmental science, and advanced materials, but several challenges must be overcome for their full implementation. One major issue is the scalability and reproducibility of nanoparticle synthesis. While precise control over their properties is achievable in the lab, ensuring consistency in large-scale production remains complex. Developing cost-effective, high-yield methods that maintain uniformity is crucial for clinical and commercial applications. Additionally, improving nanoparticle stability through optimized surface coatings and functionalization techniques will enhance circulation time and therapeutic effectiveness. Biocompatibility and long-term safety are also critical concerns. Although iron oxide-based MNPs are generally considered safe, prolonged accumulation in organs such as the liver and spleen raise toxicity risks. Researchers are addressing this by developing biodegradable and excretable formulations that retain magnetic properties while being efficiently cleared from the body. Surface modifications with biocompatible materials can further improve interactions with biological systems and reduce immune responses. Standardized toxicity assessment protocols will be essential for regulatory approval. Another promising area is the development of multifunctional MNP platforms that integrate diagnostics and therapy. Combining magnetic targeting with imaging, photothermal therapy, or enzyme-mimetic activity could revolutionize personalized medicine. Hybrid nanoparticles with both plasmonic and magnetic components are being explored for dual-modal imaging and precision-targeted treatments. Advances in artificial intelligence (AI) may further enhance real-time disease monitoring by improving MNP-based imaging and therapeutic accuracy. Beyond healthcare, MNPs are gaining attention in environmental and energy applications. Their high surface area and magnetic properties make them valuable for removing pollutants from water, while their potential in energy storage, catalysis, and electromagnetic shielding is being explored for next-generation batteries and electronics. Integrating MNPs with materials science and environmental engineering could lead to major advancements in sustainable technology. To translate these innovations into real-world applications, regulatory and clinical challenges must be addressed. Comprehensive studies on nanoparticle pharmacokinetics, long-term biodistribution, and metabolic pathways are necessary for establishing safety guidelines. Regulatory bodies require rigorous characterization protocols before approving MNP-based medical applications. Future efforts should focus on developing highly efficient, minimally toxic, and precision-targeted MNPs that meet safety and efficacy standards. Overcoming these challenges will enable the widespread adoption of MNPs in healthcare, environmental sustainability, and advanced materials.

## Conclusion

Magnetic nanoparticles are increasingly recognized as important medical tools, particularly for diagnostic and therapeutic applications. Their unique magnetic properties make them ideal contrast agents for MRI, enhancing image quality and aiding in the early detection of diseases by improving the visibility of tissues and abnormalities. SPIONs are widely used in clinical settings for liver imaging and angiography, demonstrating their effectiveness in diagnostic imaging. Additionally, MNPs enable targeted drug delivery by concentrating therapeutic agents in specific areas of the body, thereby minimizing adverse effects. This magnetic drug-targeting approach enhances treatment efficacy by delivering medications precisely to the required locations, particularly in cancer therapy. Surface coatings can further improve targeting efficiency and biocompatibility by modifying the physical and chemical properties of the particles. Additionally, magnetic thermoablation utilizes nanoparticles to generate localized heat in specific tissues, providing a novel approach for cancer treatment. These applications highlight the versatility of MNPs in advancing both medical diagnostics and therapeutic interventions, contributing to the development of more effective and minimally invasive treatments.

## Data Availability

The author declares that the data supporting the findings of this study are available within the paper.

## Abbreviations

AMF: Alternating magnetic field
DLS: Dynamic light scattering
EPR: Enhanced permeability and retention
FCC: Face-centered cubic
HD: Hydrodynamic diameter
IONPs: Iron oxide nanoparticles
MGT: Magnetic gene targeting
MHT: Magnetic hyperthermia therapy
MNPs: Magnetic nanoparticles
MPS: Mononuclear phagocyte system
MRI: Magnetic resonance imaging
NPs: Nanoparticles
ODNs: Oligodeoxynucleotides
PDT: Photodynamic therapy
PEG: Polyethylene glycol
ROS: Reactive oxygen species
SAR: Specific absorption rate
SPIONs: Superparamagnetic iron oxide nanoparticles
TEM: Transmission electron microscopy
